# Community-based palliative care is associated with reduced emergency department use by people with dementia in their last year of life: A retrospective cohort study

**DOI:** 10.1177/0269216315576309

**Published:** 2015-09

**Authors:** Lorna Rosenwax, Katrina Spilsbury, Glenn Arendts, Bev McNamara, James Semmens

**Affiliations:** 1 School of Occupational Therapy and Social Work, Faculty of Health Sciences, Curtin University, Perth, WA, Australia; 2 Centre for Population Health Research, Faculty of Health Sciences, Curtin University, Perth, WA, Australia; 3 Centre for Clinical Research in Emergency Medicine, Harry Perkins Institute of Medical Research and The University of Western Australia, Perth, WA, Australia; 4 Department of Emergency Medicine, Royal Perth Hospital, Perth, WA, Australia

**Keywords:** Palliative care, emergency service, hospital, dementia, follow-up studies

## Abstract

**Objective:**

To describe patterns in the use of hospital emergency departments in the last year of life by people who died with dementia and whether this was modified by use of community-based palliative care.

**Design:**

Retrospective population-based cohort study of people in their last year of life. Time-to-event analyses were performed using cumulative hazard functions and flexible parametric proportional hazards regression models.

**Setting/participants:**

All people living in Western Australia who died with dementia in the 2-year period 1 January 2009 to 31 December 2010 (dementia cohort; *N* = 5261). A comparative cohort of decedents without dementia who died from other conditions amenable to palliative care (*N* = 2685).

**Results:**

More than 70% of both the dementia and comparative cohorts attended hospital emergency departments in the last year of life. Only 6% of the dementia cohort used community-based palliative care compared to 26% of the comparative cohort. Decedents with dementia who were not receiving community-based palliative care attended hospital emergency departments more frequently than people receiving community-based palliative care. The magnitude of the increased rate of emergency department visits varied over the last year of life from 1.4 (95% confidence interval: 1.1–1.9) times more often in the first 3 months of follow-up to 6.7 (95% confidence interval: 4.7–9.6) times more frequently in the weeks immediately preceding death.

**Conclusions:**

Community-based palliative care of people who die with or of dementia is relatively infrequent but associated with significant reductions in hospital emergency department use in the last year of life.

**What is already known about the topic?**

- Cancer patients are known to visit emergency departments (EDs) less frequently when they receive community-based palliative care.
- People with dementia are commonly seen in EDs.
- It is not known whether provision of community-based palliative care will reduce ED use in patients dying with dementia.

**What this paper adds?**

- A total of 6% of people dying with dementia in Western Australia received community-based palliative care in the last year of life compared to 26% of the comparative group of decedents.
- In their last year of life, people with dementia who were not receiving community-based palliative care visited EDs up to six times more frequently than people with dementia who were receiving community-based palliative care.

**Implications for practice, theory or policy**

- Increasing access to community-based palliative care is likely to reduce the demand on EDs by people with dementia.

## Introduction

Emergency departments (EDs) regularly care for people with life-limiting chronic disease. Visits to the ED by people with advanced chronic disease near the end of life can cause distress and exhaustion for patients and their families, while being clinically challenging for staff.^1^ There have been initiatives to integrate palliative care into the ED setting such as the US-based Improving Palliative Care in Emergency Medicine project that provides resources to assist EDs to identify and manage palliative care patients.^2^ However, there is an argument that the use of EDs towards the end of life is an indicator of poor-quality care in cancer patients^3^ and the same may be suggested for people dying with dementia.

People with dementia commonly present to ED.^4,5^ When placed in an ED setting, a person with dementia is more likely to experience distressing secondary complications such as delirium^6^ and unnecessary invasive procedures.^7^ However, further detailed research is needed to fully understand ED service use by people with dementia in their last year of life before effective, acceptable and economically viable alternatives can be provided.

Improved access to community-based palliative care for persons with dementia is one such alternative. A recent Western Australian study reported that community-based palliative care in patients with cancer reduced the number of ED visits in the last year of life.^8^ It is not clear whether this would be the case for people living with dementia given the different and often challenging underlying disease process associated with the disease. People with dementia are less likely to receive both hospital and community care in the last year of life.^9^ Nearly two-thirds of all admissions to hospital for people with dementia were emergency admissions and 16% of the total bed-days in the last year of life were due to problems accessing alternative medical facilities or related to care provider dependency.^10^ This population-based study aimed to describe patterns in the use of ED by people who had dementia in their last year of life and determine whether this was modified by the use of community-based palliative care services.

## Methods

### Study design

This was a retrospective cohort study of the last year of life of persons with dementia and who died in Western Australia (WA). WA is the largest state of Australia (2.5 million km^2^) with a population of 2.5 million that is mostly concentrated in the south-west corner of the state. A pool of decedents aged 20 years and greater who had a death registration record in WA from 1 January 2009 to 31 December 2010 was created. Decedents with the underlying cause of death coded (International Classification of Diseases, Tenth Revision, Australian Modification (ICD-10-AM) codes^11^) as related to pregnancy and the perinatal period (O.00-P.98) or injury or poisoning (S00-T98) were excluded.

A de-identified extraction from the WA Data Linkage System^12^ of each decedent’s linked death registration, hospital discharge records, ED visits, mental health outpatient visits and community-based care services data in the last year of life was provided by the Data Linkage Branch of the Health Department WA. Approval was granted by Curtin University and the Health Department WA Human Research Ethics Committees.

### Cohort definitions

From the pool of decedents, the dementia cohort was defined by having ICD-10-AM dementia-specific codes anywhere within the death certificate. The codes used were G30 (Alzheimer’s disease), F01 (Vascular dementia), F02 (Dementia in diseases including Creutzfeldt–Jakob disease, Huntington’s disease, Parkinson’s disease and HIV/AIDS), G31.0 (Pick’s disease, fronto-temporal dementia), G31.3 (Lewy Body disease), F03 (Unspecified dementia), A81.0 (Creutzfeldt–Jakob disease) and G10 (Huntington’s disease). Additional decedents were included if there was any mention of the above dementia codes in hospital records, mental health outpatient visits or ED visits in the last year of life, even if they were not recorded on the death certificate.

A comparative cohort was also identified as a decedent not already in the dementia cohort who had a principal cause of death on Part 1 of the death certificate listed as due to neoplasms, heart failure, renal failure, liver failure, chronic obstructive pulmonary disease, motor neurone disease or HIV/AIDS. These conditions have been described previously as amenable for palliative care services in the WA context.^13^ The comparative cohort was randomly chosen using a uniform distribution such that the final age (5-year age groups) and sex distribution were similar to those of the dementia cohort.

### Variable definitions

Service dates from Silver Chain WA data were used to identify the time spent receiving community-based palliative care. Silver Chain WA is a not-for-profit organisation that provides over 90% of in-home health and care. Community-palliative care was defined as palliative care provided by Silver Chain at the place of usual residence, whether a private residence or care facility. Palliative care is only provided with a referral from a medical practitioner and includes at-home physical care and practical support, symptom management (e.g. pain, nausea), counselling, respite option and links to other community and government services.^14^

Data for each ED visits in the last year of life for the two decedent cohorts included the triage category and hospital admission status. Coded presenting symptom information was available for major metropolitan public hospitals only (approximately 80% of ED visits). Residential address at the time of death and during hospital stays and ED visits were classified as private residence; residential aged care facility (RACF); or other care facilities such as hospital, hospice or other/unknown. Marital status at the time of death was classified as partnered or non-partnered. Accessibility to service categories was based on the Accessibility and Remoteness Index of Australia (ARIA+).^15^ Comorbidity was defined using Elixhauser binary indicators from conditions recorded during in-patient hospitals stays.^16^ Comorbidity could only be estimated for decedents who had a hospital stay in the last year of life and could therefore be under-ascertained.

### Statistical analysis

Pearson’s chi-squared tests assessed equality of proportions between the dementia and comparative cohorts. For time-to-event analysis, data were in the counting process format whereby each day in the last year of life of each individual decedent in the dementia and comparative cohorts was assigned to one of four possible residential care states: (1) regular care while living in private residence, (2) regular care while living in a care facility (RACF or other), (3) community-based palliative care and (4) in-patient hospital care. If an ED visit occurred on the same day a new residential care state began, the ED visit was presumed to have preceded the change in residence. Residential address at the time of each hospital stay and ED visits was carried backwards in time until the next previous hospital stay, ED visit or until start of follow-up (Day 1) if no earlier service records were available. Thus, a decedent who visited ED on Day 60 while living in a private residence and on Day 200 while living in a RACF was considered to have been living in a private residence Days 1–60 and a care facility from Day 61 to 200. The residence at the time of death was assigned to decedents with no ED or hospital stays in the last year of life. Any visit to an ED was considered a failure event and multiple failures were possible for each decedent. Multiple visits to ED on the same day by the same decedent were considered as a single failure event. A decedent was not considered to be at risk of an ED visit during any hospital stays.

The Nelson–Aalen cumulative hazard function was used to depict the cumulative number of ED visits over the last year of life. Flexible parametric proportional hazards survival regression models^17^ were constructed to investigate the association of ED use and community-based palliative care for the dementia cohort after taking other predictors for ED use into account. Due to non-proportional hazards, the residential care state variable was entered into the model as a time-dependent spline function with three knots. Robust variance estimators were used to adjust standard errors to account for clustering. All analyses were performed using Stata 13.^18^

## Results

The dementia cohort comprised 5261 decedents. The majority (3603; 68%) were identified from death certificate data with a further 30% (*n* = 1595) identified from hospital discharge data, 0.5% (*n* = 25) from ED visits and 0.7% (*n* = 38) from mental health outpatient records in that order. The median age at death was 87 years (range 21–107 years). Females comprised 61% of the cohort. Most of the dementia cohort (*n* = 3433; 65%) was recorded as having unspecified dementia. There were 1333 (25%) decedents with Alzheimer’s disease, 376 (7%) with vascular dementia and 119 (2%) with dementia in other diseases. The dementia in other disease category included Pick’s disease (*n* ⩽ 5), Creutzfeldt–Jakob disease (*n* = 14), Huntington’s disease (*n* = 21), dementia in Parkinson’s disease (*n* = 45), dementia in HIV/AIDS (*n* ⩽ 5) and Lewy body disease and other diseases (*n* = 36).

The comparative cohort comprised 2865 decedents. The age and sex distribution of the comparative cohort was similar to the dementia cohort as was the distribution of Aboriginal and/or Torres Strait Islander status and marital status (Table 1). However, decedents with dementia were much more likely to have lived in a RACF (63% vs 28%), in a major city and much less likely to have attended a hospital in the last year of life relative to the comparison cohort.

**Table 1.** Summary characteristics of the dementia and comparative decedent cohorts.

	Dementia cohort, *N* = 5261		Comparative cohort, *N* = 2865		*χ*^2^
	*n*	%	*n*	%	*p* value
Age at death (years)					
<60	43	0.8	24	0.8	0.968
60–69	127	2.4	70	2.4	
70–79	710	13.5	389	13.6	
80–89	2652	50.4	1456	50.8	
90–99	1642	31.2	886	30.9	
⩾100	87	1.7	40	1.4	
Sex					
Male	2077	39.5	1138	39.7	0.831
Female	3184	60.5	1727	60.3	
Marital status					
Non-partnered/unknown	3570	67.9	1913	66.8	0.317
Partnered	1691	32.1	952	33.2	
Aboriginal and/or TSI					
No or unknown	5,203	98.9	2,841	99.2	0.253
Aboriginal and/or TSI	58	1.1	24	0.8	
Residence at the time of death					
Private residence	1644	32.5	1978	69.5	<0.001
RACF	3,203	63.2	795	27.9	
Other care facilities	191	3.8	53	1.9	
Unknown/other	24	0.5	20	0.7	
Place of death					
Private residence	222	4.4	494	17.4	<0.001
Hospital	1542	30.4	1438	50.5	
RACF	3026	59.7	729	25.6	
Other care facility	266	5.3	174	6.1	
Unknown/other	10	0.2	11	0.4	
ARIA+					
Major cities	4007	76.2	2069	72.2	0.003
Inner regional	717	13.6	450	15.7	
Outer regional	375	7.1	248	8.7	
Remote	107	2.0	68	2.4	
Very remote	50	1.0	24	0.8	
In the last year of life					
Any hospital stay					
No	1592	30.3	375	13.1	<0.001
Yes	3669	69.7	2490	86.9	
Any community-based palliative care					
No	4964	94.4	2124	74.1	<0.001
Yes	297	5.6	741	25.9	
Any community-based care^a^					
No	4005	76.1	1375	48.0	<0.001
Yes	1256	23.9	1490	52.0	

TSI: Torres Strait Islander; RACF: residential aged care facility; ARIA+: Accessibility and Remoteness Index of Australia.

a Includes home help, meals, personal care and respite.

**Figure 1.**
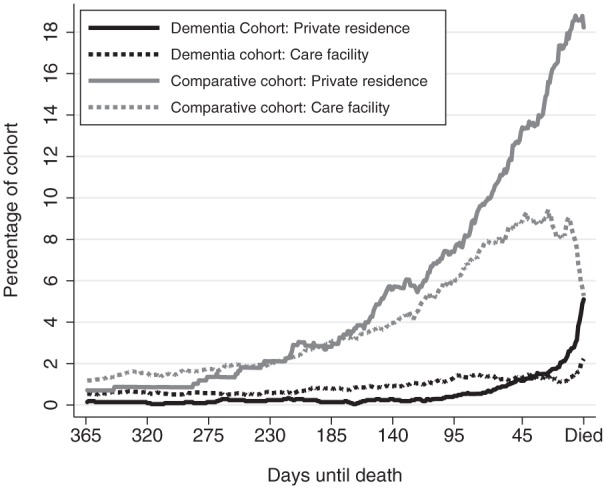
The proportion of the dementia and comparative cohorts receiving community-based palliative care each day in the last year of life for people living in private residences and people living in care facilities, which includes both residential aged care facilities and other facilities.

Overall, 6% of the dementia cohort used community-based palliative care in the last year of life compared to 26% of the comparative cohort. The use of community-based palliative care varied markedly over the last year of life with the proportion of the dementia and comparative cohorts receiving community-based palliative care increasing closer to the date of death (Figure 1). Use of palliative care services by the comparative cohort increased much earlier in the last year of life compared to the dementia decedents who used this service. Use of community-based palliative care was more prevalent in decedents who were living in private residences compared to those living in care facilities, regardless of cohort.

### ED use in the last year of life

More than 70% of decedents in both the dementia and comparative cohorts attended an ED at least once in the last year of life (Table 2). The comparative cohort also had a greater number of days visiting EDs compared to the dementia cohort, particularly in the days closer to death. Visits to the ED by the dementia cohort tended towards being triaged as less urgent although 3.6% of the dementia cohort were categorised as requiring resuscitation compared to 2.9% of the comparative cohort. The dementia cohort had a higher proportion of neurological and mental disorder presenting symptoms and a higher proportion of fall- and injury-related symptoms at presentation to ED. In contrast, the comparative cohort had more cardiac and abdominal pain-related presentations to ED.

**Table 2.** Characteristics of ED use in the last year of life for the dementia and comparative cohorts.

In last year of life	Dementia cohort, *N* = 5261		Comparative cohort, *N* = 2865		*χ*^2^
	*n*	%	*n*	%	*p* value
Any ED visit					
No	1421	27.0	679	23.7	0.001
Yes	3840	73.0	2186	76.3	
Median number of ED visits (IQR)	1	0–3	1	1–3	
Mean number of ED visits (SD)	1.9	2.1	2.0	2.3	
Range of number of ED visits; min. and max.	0	43	0	29	
Total number of days in ED (% of all days)	9781	0.5	5827	0.6	<0.001
Hospital admissions from ED	6665	68.1	4112	70.6	0.002
Days in ED by quarters of last year of life					
Days 1–91	1470	0.3	763	0.3	0.281
Days 92–181	1658	0.4	973	0.4	0.064
Days 182–273	1961	0.4	1261	0.5	<0.001
Days 274–death	4692	1.0	2830	1.1	<0.001
Triage category					
Resuscitation: immediate	349	3.6	167	2.9	<0.001
Emergency: within 10 min	1622	16.6	1132	19.4	
Urgent: within 30 min	4079	41.7	2502	42.9	
Semi-urgent: within 60 min	3399	34.8	1739	29.8	
Non-urgent: within 120 min	293	3.0	247	4.2	
Other	39	0.4	39	0.7	
Number and proportion of ED visits that resulted in hospital admission by triage category					
Resuscitation: immediate	279	79.9	136	81.4	0.689
Emergency: within 10 min	1335	82.1	956	84.5	0.138
Urgent: within 30 min	3001	73.6	1925	76.9	0.002
Semi-urgent: within 60 min	1922	56.6	1004	57.7	0.416
Non-urgent: within 120 min	94	32.1	57	23.1	0.020
10 most frequent ED presenting symptoms^a^ for dementia cohort					
Shortness of breath	711	9.0	707	16.4	<0.001
Lower leg injury	526	6.7	134	3.1	
Altered conscious state	410	5.2	67	1.6	
Chest pain	375	4.8	393	9.1	
Head injury (includes closed)	305	3.9	62	1.4	
Confusion/altered mental state	299	3.8	74	1.7	
Abdominal pain	259	3.3	277	6.4	
Collapse	247	3.1	116	2.7	
Fall	234	3.0	72	1.7	
Nausea/vomiting	134	1.7	99	2.3	

ED: emergency department; IQR: inter-quartile range; SD: standard deviation.

a Symptom data were only available for 80% of ED visits due to hospital coding practices.

**Figure 2.**
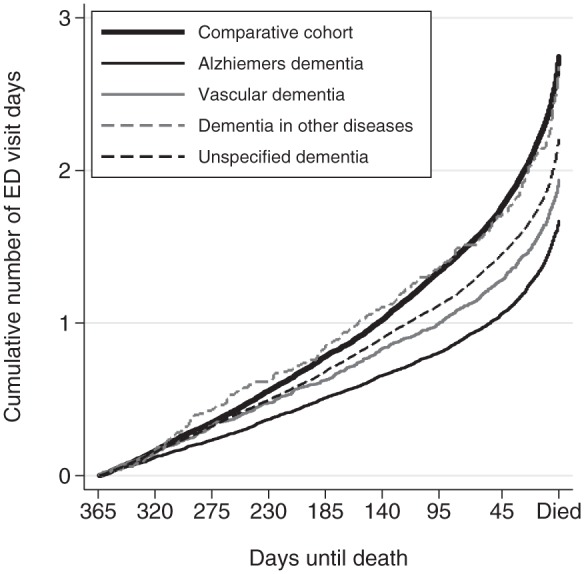
The average cumulative number of days with ED visits in the last year of life for decedents with different dementia subtypes and the overall comparative cohort (Nelson–Aalen cumulative hazard function).

To determine whether ED use varied by the type of dementia, the cumulative hazard (number of days) of visiting ED was estimated for the dementia cohort subtypes and the comparative cohort (Figure 2). The pattern of cumulative number of days visiting ED for decedents with dementia in other diseases was similar to the comparative cohort. At the time of death, decedents with dementia in other disease attended ED on average of 2.7 (95% confidence interval (CI): 2.4–3.0) days compared to 2.7 (95% CI: 2.6–2.8) days for the comparative cohort. For the other types of dementia, the rate of visiting ED was much lower with decedents with Alzheimer’s dementia on average making 1.7 (95% CI: 1.6–1.7) visits to ED in the last year of life.

**Figure 3.**
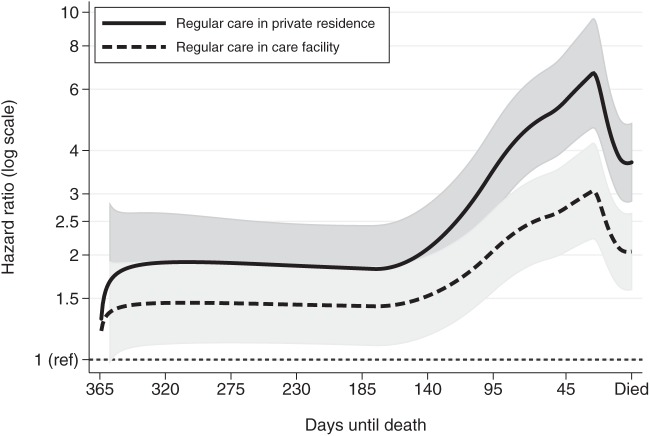
Relative hazard (rate) of daily ED visits for decedents with dementia who were not receiving palliative care and living in a private residence or a care facility compared to decedents with dementia who were receiving community-based palliative care (reference rate HR of 1 – constant thin dashed line at bottom of graph) over the last year of life. Predicted hazard ratios are from the same flexible parametric proportional hazard model in Table 3. Due to non-proportional hazards over the last year of life, the type of care variable was entered into the model as a time-varying covariate. Grey shading represents 95% CI of estimated hazard ratio.

Flexible parametric survival models were used to estimate the impact of community-based palliative care on ED use by the dementia cohort and whether this was modified by other factors or varied over time. The rate of ED visits was significantly increased when not receiving community-based palliative care, and this increase varied markedly over the last year of life (Figure 3). For the first 130 days of the last year of life, those receiving regular care in a private residence visited ED almost twice as often as those receiving palliative care (Hazard Ratio 1.9; 95% CI: 1.4–2.5). Decedents with dementia who received regular care in a care facility visited the ED 1.4 times more often than those receiving community-based palliative care (95% CI: 1.1–1.9). In the last month of life, the relative rate of ED visits for those receiving regular care jumped markedly compared to those receiving palliative care reaching a maximum rate around day 340 or 25 days before death. Those receiving regular care in private residences visited EDs 6.7 (95% CI: 4.7–9.6) times more frequently and those receiving regular care in a care facility visited EDs 3.1 (95% CI: 2.2–4.2) times more frequently than those of dementia cohort who were receiving palliative care at that time.

Other factors associated with increased ED use in the dementia cohort were being male, being younger and living with dementia with other diseases rather than Alzheimer’s or vascular dementia, living in outer regional and remote areas and having certain types of comorbid conditions (Table 3). Decedents with dementia who were partnered at the time of death also had increased rates of ED visits as did decedents who had a prior history of ED visits.

**Table 3.** Factors associated with daily rate of ED use in the last year of life in the dementia cohort as estimated from a flexible parametric proportional hazards model (*N* = 5261).

	Hazard ratio	95% CI	*p* value
Age at death (years)			
<60	1.20	0.95–1.51	0.126
60–69	1.01	0.86–1.17	0.883
70–79	1.07	099–1.15	0.080
80–89	1	Ref	
90–99	0.87	0.82–0.92	<0.001
⩾100	0.71	0.55–0.89	0.003
Sex			
Female	1	Ref	
Male	1.16	1.10–1.23	<0.001
Marital status			
Non-partnered/unknown	1	Ref	
Partnered	1.10	1.0–1.2	0.001
Type of dementia			
Alzheimer’s dementia	1	Ref	
Vascular dementia	1.08	0.98–1.19	0.105
Dementia in other diseases	1.28	1.10–1.49	0.002
Dementia unspecified	1.18	1.10–1.25	<0.001
Residential care state^a^			
Regular care not in a care facility			
Regular care in a care facility	See Figure 3 and text		
Community-based palliative care			
ARIA+			
Major cities	1	ref	
Inner regional	1.03	0.95–1.11	0.417
Outer regional	1.23	1.13–1.36	<0.001
Remote	1.30	1.07–1.57	0.008
Very remote	0.95	0.74–1.21	0.663
Year of death			
2009	1	Ref	
2010	0.95	0.90–0.99	0.027
Number of previous ED visits in year	1.12	1.07–1.16	<0.001
Comorbidity in last year of life (yes/no)^b^			
Peptic ulcer disease	1.62	1.36–1.92	<0.001
Malignancies	1.66	1.49–1.83	<0.001
Hypertension	2.63	2.00–3.45	<0.001
Chronic pulmonary disease	1.51	1.33–1.71	<0.001

ED: emergency department; CI: confidence interval; ARIA: Accessibility and Remoteness Index of Australia.

a Showed non-proportional hazards over last year of life and was entered as an interaction with time (see Figure 3 and text in ‘Results’ section).

b Only comorbidities with a hazard ratio greater than 1.5 were included in final regression model.

## Discussion

In this study of all Western Australian people who died with or of dementia in 2009–2010, we found that the provision of community-based palliative care was associated with a significantly reduced daily rate of ED visits over their last year of life. As less than 6% of the dementia cohort received community-based palliative care at any one time over the last year of life, there is vast potential for increasing access to palliative services in this group of persons and thus reducing attendances at EDs. While palliative care has been shown to be associated with reduced ED use by cancer patients, this is the first study to show a similar reduction for dementia, despite the reasons for attending EDs being different.

A low rate of palliative care provision in people with dementia, as found in our study, has several origins. Modern palliative care has its longest period of association with cancer medicine,^19^ and clinicians frequently do not perceive terminal illness that is not cancer related as being amenable to palliative care.^20^ Particular difficulties arise with dementia which is not widely understood in the broader community to be a life-limiting illness.^21^ There is also a paucity of high-level evidence to guide referrals to palliative care services for people with dementia,^22^ so even when it is recognised as a terminal illness, there may be indecision surrounding palliative care commencement. Finally, palliative care provision may be unfeasible in some patients with advanced dementia, necessitating a validated symptom assessment scale for this population. Such a tool would incorporate a validated functional assessment scale to guide practitioners in deciding whether a dementia patient is in the terminal stages of their disease.^23^

The proportion of decedents living in a care facility who also received community-based palliative care in this study was low. Palliative and hospice care for people dying in nursing homes is relatively uncommon, yet highly valued and associated with improved symptom management and reduced hospitalisations in US studies.^24,25^ Particular barriers to the provision of palliative care in RACF have been well documented, though most research on end-of-life care in RACF is also observational and biased towards describing poor care.^26,27^ Adoption of palliative care is often slowed by indecision and episodic transfer to hospital for acute care until the last days of life,^28^ and there are a number of logistical barriers related to staff training, bureaucracy and staff–family conflict that influence end-of-life care in RACF.^29^ There is a strong evidence base and policy focus on advance care planning in RACF,^30^ but this is a strategy that is complementary to but not necessarily inclusive of palliative care. It is clear that strategies to increase uptake of palliative care in patients with dementia must include measures for the special circumstances of people living in RACF.

We have found that the proportion of people who use ED in the last year of life is similar in populations with and without dementia, but patients with dementia have different leading causes for presentations, with injuries and falls being relatively more prevalent in the dementia group. Although the association between dementia, frailty and injury is well known, it is also possible that this difference represents poor recognition of some symptoms by the carers of people with dementia, particularly non-injurious pain.^31^

Previous qualitative research has highlighted the ‘under-triaging’ of older people with dementia in ED, meaning patients are on average given lower triage urgency scores for the same illness acuity.^32^ Our results support this finding, with people with dementia receiving lower triage urgency scores and having higher rates of admission in the lower (triage scale 4 and 5) triage categories. Under-triage is one of a number of deficiencies highlighted in the care of older people with dementia in ED^33^ as a body of evidence builds that the ED environment is unsuitable for the needs of vulnerable older people.^34^

There are several limitations to our study. This was a retrospective study using administrative data that were not collected specifically for this research purpose, and as such, it was not possible to draw casual inferences. This limitation could be overcome using a randomised experimental design, although it may be difficult or unethical to conduct in a setting of cognitive impairment. Other limitations were clinical detail such as the severity of cognitive impairment was lacking. Coding deficiencies may have resulted in comorbidity being under-reported, and this could particularly occur in people with dementia due to problems with history taking. Dementia case under-ascertainment from relying on administrative data was likely to have introduced some misclassification bias. Finally, we were not able to accurately define where a decedent was living over the whole of the last year of life, only where they died.

## Conclusion

In conclusion, community-based palliative care in people who die with or of dementia is relatively infrequent but associated with significant reductions in ED use in the last year of life. There is a need to develop a strong evidence base for palliative care provision in this population and cost-effective models for care delivery.
